# Phage Display of the Serpin Alpha-1 Proteinase Inhibitor Randomized at Consecutive Residues in the Reactive Centre Loop and Biopanned with or without Thrombin

**DOI:** 10.1371/journal.pone.0084491

**Published:** 2014-01-10

**Authors:** Benjamin M. Scott, Wadim L. Matochko, Richard F. Gierczak, Varsha Bhakta, Ratmir Derda, William P. Sheffield

**Affiliations:** 1 Department of Pathology and Molecular Medicine, McMaster University, Hamilton, Ontario, Canada; 2 Department of Chemistry, Alberta Glycomics Centre, University of Alberta, Edmonton, Alberta, Canada; 3 Canadian Blood Services, Research and Development, Hamilton, Ontario, Canada; University of Bern, Switzerland

## Abstract

In spite of the power of phage display technology to identify variant proteins with novel properties in large libraries, it has only been previously applied to one member of the serpin superfamily. Here we describe phage display of human alpha-1 proteinase inhibitor (API) in a T7 bacteriophage system. API M358R fused to the C-terminus of T7 capsid protein 10B was directly shown to form denaturation-resistant complexes with thrombin by electrophoresis and immunoblotting following exposure of intact phages to thrombin. We therefore developed a biopanning protocol in which thrombin-reactive phages were selected using biotinylated anti-thrombin antibodies and streptavidin-coated magnetic beads. A library consisting of displayed API randomized at residues 357 and 358 (P2–P1) yielded predominantly Pro-Arg at these positions after five rounds of thrombin selection; in contrast the same degree of mock selection yielded only non-functional variants. A more diverse library of API M358R randomized at residues 352–356 (P7–P3) was also probed, yielding numerous variants fitting a loose consensus of DLTVS as judged by sequencing of the inserts of plaque-purified phages. The thrombin-selected sequences were transferred en masse into bacterial expression plasmids, and lysates from individual colonies were screening for API-thrombin complexing. The most active candidates from this sixth round of screening contained DITMA and AAFVS at P7–P3 and inhibited thrombin 2.1-fold more rapidly than API M358R with no change in reaction stoichiometry. Deep sequencing using the Ion Torrent platform confirmed that over 800 sequences were significantly enriched in the thrombin-panned versus naïve phage display library, including some detected using the combined phage display/bacterial lysate screening approach. Our results show that API joins Plasminogen Activator Inhibitor-1 (PAI-1) as a serpin amenable to phage display and suggest the utility of this approach for the selection of “designer serpins” with novel reactivity and/or specificity.

## Introduction

The serpin superfamily contains within it many proteins that regulate biologically important processes (reviewed in [1], [2]). Regulation is achieved by inhibition of many proteinases that mediate coagulation, fibrinolysis, inflammation, and complement fixation, to name a few of the physiological processes in which serpins play key roles [3]. Although serpins have been discovered that inhibit, for instance, cysteine proteinases [4], the original acronym (serine proteinase inhibitors) [5] remains a useful descriptor for many human serpins with known serine proteinase targets [6]. Serpins exhibit a conserved three-dimensional structure comprised of eight or nine alpha helices, three beta sheets, and a surface loop protruding from the body of the inhibitor called the Reactive Centre Loop (RCL) [7]. Following formation of an encounter complex between proteinase and serpin and cleavage of the scissile bond (the reactive centre) a dramatic conformational change takes place; bond cleavage releases energy stored within the serpin fold, powering translocation of the proteinase to the opposite pole of the serpin and the RCL inserts into an underlying beta sheet in the serpin body as a beta strand [8]. The proteinase ends up trapped by an acyl bond tethering it to the cleaved serpin and by the distortion of its active site, which prevents it from completing its catalytic cycle [9], [10].

Although molecular and structural discoveries have greatly advanced our knowledge of the serpin mechanism over the last few decades, our understanding remains incomplete with respect to the key role of the RCL. The RCL extends for 20–24 residues in most serpins [7]. While the reactive centre, termed by convention the P1–P1′ bond [11], is important in serpin activity and specificity, natural and engineered mutations in the RCL at either N-terminal (numbered P2, P3, etc.) or C-terminal (numbered P1′, P2′, etc.) positions in the RCL have been shown to affect both parameters [12]. In general, alignments of the RCL across the serpin family show little conservation for most RCL residues [3]. Efforts to alter serpin specificity by mutagenesis of RCL residues have met with some success [13], [14], but more often the resulting inhibitors are handicapped by a decreased stability and/or increased propensity to act as proteinase substrates, rather than proteinase inhibitors [15]–[22].

Alpha-1 proteinase inhibitor (API, also known as alpha-1 antitrypsin) has been used as a scaffold for serpin mutagenesis and engineering studies since a rare mutation of its P1 residue from methionine to arginine (M358R) was observed to re-orient API from inhibiting elastase to inhibiting the key coagulation proteinase, thrombin [23]. In its new role, the mutated inhibitor was seemingly so effective at thrombin inhibition that the affected patient succumbed to a bleeding disorder [23]. Pre-clinical studies demonstrated further refinements in specificity and activity were required prior to contemplating this protein as a therapeutic agent in individuals prone to overactive coagulation [24]–[26]. Both our laboratory and other groups have made some progress in this regard by using mutagenesis strategies in which additional residues from other serpins were substituted into the API M358R [27]–[30].

A more exhaustive and ideal way of systematically probing the contribution of RCL residues to serpin activity and/or specificity would be to generate large expression libraries of serpins with hypervariable RCLs and probe them with different proteinases. Expression of the library on the surface of cells or viruses encoding the library, and the ability to select such entities for proteinase binding, would link affinity selection to the DNA encoding the variants. Phage display is one technique that fits these criteria [31]; to date, however, it has only been applied to studies of one of the 36 human serpins, plasminogen activator inhibitor -1 (PAI-1), and variability was randomly introduced using error-prone PCR [32]–[35]. M13 phagemid-based [32]–[35] or bacteriophage lambda-based [36] vectors were employed. With only a single serpin having been reported in the phage display literature, it was not known if special characteristics of PAI-1 rendered it particularly amenable to the technique, or if it could be more generally applied to other serpins. Our aims in the current study were therefore: to establish that API displayed on the surface of T7 bacteriophages retained the ability to covalently bind thrombin; to construct libraries of displayed API variant in up to 5 consecutive RCL residues; to use them to identify novel API variants with thrombin-inhibitory activity; and to assess if any of the identified variants demonstrated increased rates of reaction compared to API M358R. We report positive findings with respect to the first three aims using phage display, and the detection of two novel API M358R variants with increased rates of thrombin inhibition relative to the “parental” recombinant protein, API M358R, among the thrombin-inhibitory variants selected by phage display.

## Materials and Methods

### Cloning API-containing inserts into T7 phage

**Figure 1:**
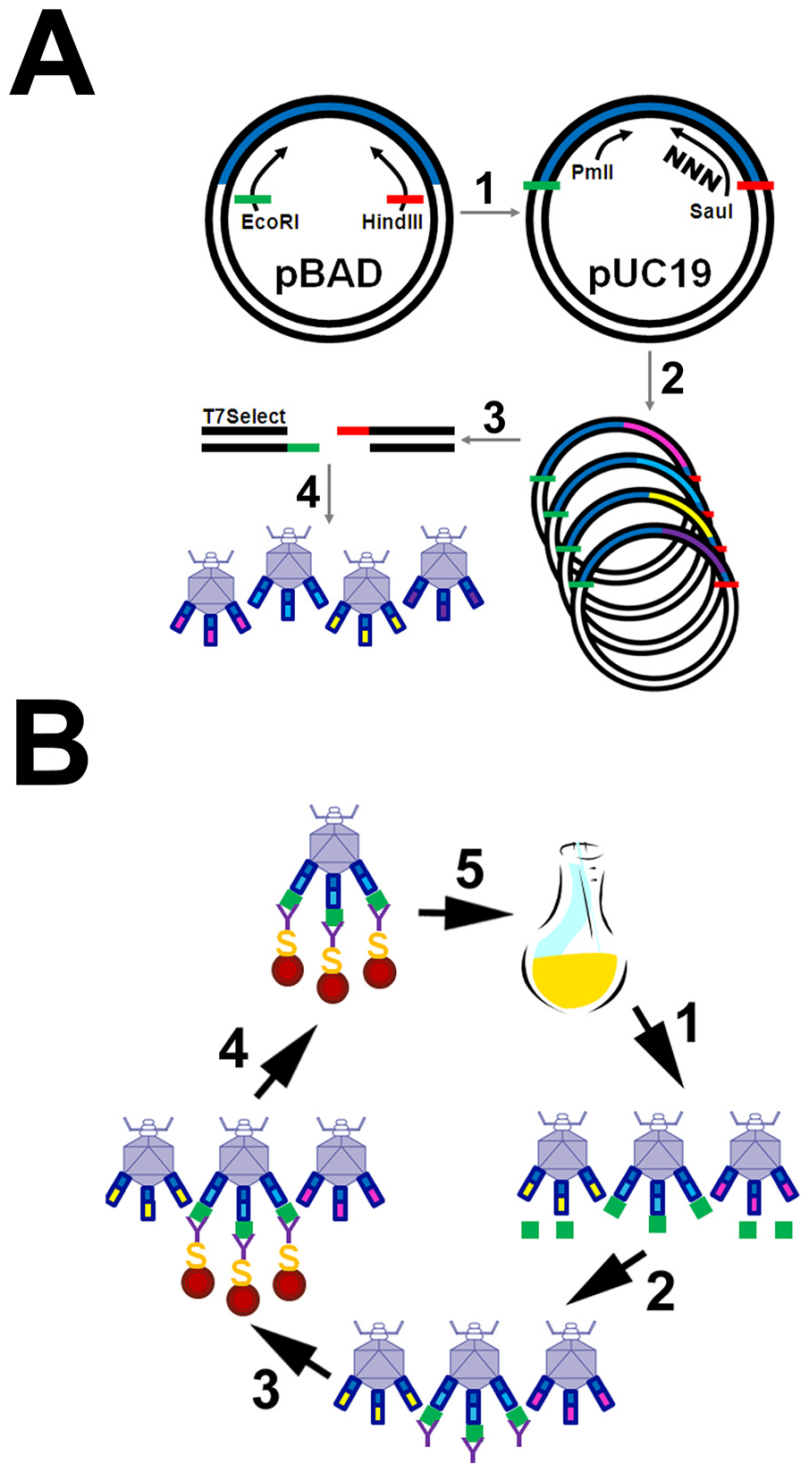
Schematic diagrams of construction of phage display library and biopanning strategy. Panel A shows the cloning strategy employed to create T7Select10-3b phage constructs displaying API M358R or libraries displaying API with randomized RCL codons. 1) The API M358R cDNA was mobilized from pBAD-H_6_API M358R [29] by PCR that appropriately positioned EcoRI (green) and HindIII cohesive ends, and ensured that it was in-frame for subsequent insertion into the T7 Select 10-3b vector, in plasmid pUC19-API M358R. 2) Degeneracy was introduced at either P2–P1 or P7–P3 codons using a sense primer overlapping the unique PmlI site or a degenerate antisense (NNN primer) overlapping the unique SauI site. PCR products were inserted into phosphatase-treated PmlI- and SauI-restricted pUC19-API M358R and the resulting plasmid library biologically amplified in E. coli TOP10. 3) EcoRI-and HindIII-restricted total digestion products of the P2–P1 or P7–P3 plasmid libraries, respectively, were then ligated to T7Select10-3b vector arms. 4) The recombinant T7Select10-3b library, containing either the API P2–P1 randomized or the API M358R P7–P3 randomized inserts, was then packaged into phages to create the respective phage display libraries. Pink, light blue, yellow, and purple API-encoding inserts (between steps 2 and 3) indicate the encoding of different variants, and the correspondingly coloured phages (step 4) represent their displayed products. Panel B shows the biopanning procedure. 1) A phage lysate produced by infection of E. coli BLT5615 with a T7Select10-3b API library was reacted with thrombin in solution (green squares), which bound to some displayed sequences (e.g. blue but not yellow or pink). 2) A biotinylated antibody specific to thrombin (Y-shaped symbols) was added to the thrombin-phages mixture, reacting with thrombin bound to phages via API-thrombin serpin-enzyme complexes. 3) Streptavidin-coated magnetic beads (yellow S shown on red circular symbols) were added to the mixture, reacting preferentially with antibody-thrombin-phage complexes. 4) Magnetic bead-streptavidin-thrombin-phage complexes were concentrated using a magnet. 5) After washing, bead assemblies were used to directly infect a fresh culture of E. coli BLT5615 to start the next round of biopanning.

Previously described bacterial expression plasmid pBAD-H_6_API (M358R) [29] was used as the template for PCR to prepare the API M358R cDNA for insertion into T7 phages, using oligonucleotide primers 5918 (5′-GATCCGAATTC AGAGGATCCC CAGGGAGATG CT-3′) and 5919 (5′-GCTAAGCTTC ATTTTTGGGT GGGATTCACC AC-3′) and heat-stable HotStar HiFidelity DNA polymerase, as directed by manufacturer (EMD Millipore, Billerica, MA). The resulting PCR product was restricted with EcoRI and HindIII and inserted between the corresponding sites of pUC19, forming pUC19-API M358R. The 1205 bp EcoRI-HindIII restriction fragment of pUC19-API M358R was gel-purified and ligated to compatible T7Select10-3b vector arms using T4 DNA ligase (Thermo Fisher Scientific, Burlington, ON), and packaged using the T7Select packaging extract, following the T7Select System manufacturer's directions (Novagen, Madison, WI), forming recombinant bacteriophage T7Select10-3b API M358R. Identical steps were followed using a control DNA insert provided by the manufacturer, encoding a 15 amino acid S-tag, forming recombinant bacteriophage T7Select10-3b S-tag. Analogous steps were taken to form two API phage libraries: one hypervariable at residues 357 – 358 (P2–P1); and the other hypervariable at residues 352–356 (P7–P3), as shown schematically (Figure 1A). In both cases, PCR on pBAD-H_6_API (M358R) was employed, and the PCR product was restricted with PmlI and SauI and inserted between these unique sites within the API cDNA in pUC19-API M358R. PmlI-SauI restricted pUC19-API M358R was treated with calf intestinal alkaline phosphatase (New England Biolabs, Pickering, ON) to preclude self-ligation of any singly cut molecules. These intermediate plasmid libraries were then mobilized en masse for ligation into T7Select10-3b with EcoRI and HindIII, forming T7Select10-3b API (P2-P1ran) and T7Select10-3b API (P7-P3ran), where “ran” indicates “randomized”. Primers 4980 (5′- CAAGGACACC GAGGAAGAGG ACTT-3′) and 5968 (5′ CTTGACCTCA GGTGGGATAG ANNNNNNTAT GGCCTCTAAA AAC-3′) and primer 4980 and primer 0201 (5′-TGACCTCAGG CGGGATAGAT CTGGGNNNNN NNNNNNNNNN CATGGCCCCA GCAGCTTC-3′) were employed in the specific PCR steps for each library, respectively, where N indicates any nucleotide. Note that the codon for Pro361 was deliberately altered to CCA in randomizing P2P1 and CCG in randomizing P7–P3, to allow easy detection of any contamination that might have arisen from API M358R constructs previously or concurrently used in the laboratory, which have the wild-type (CCC) codon usage. Phage titering by plaque assays and amplification via either plaque or liquid lysate methods was conducted using *E. coli* BLT5615, following the manufacturer's instructions.

### Reaction of purified phages with thrombin

T7Select10-3b API M358R and T7Select10-3b S-tag phages were separately purified by combining phage lysates in Luria Broth with 1/6 volume of 50% (v/v) polyethylene glycol 8000, incubating on ice for 2 hours, and centrifuging the suspension at 4°C for 30 minutes at 10,000 X g in a Sorvall RC5B Plus centrifuge. The drained pellet was resuspended in 1.0 M NaCl, 10 mM Tris-Cl pH 8.0, 1 mM disodium EDTA. 10^10^ plaque forming units (pfu) of each library was diluted into phosphate-buffered saline (PBS) in the presence or absence of 20 nM human α-thrombin (Enzyme Research Laboratories, South Bend, IN). Reactions were analyzed by SDS-PAGE under reducing conditions, followed by immunoblotting. Immunoblots were probed with sheep anti-prothrombin affinity-purified IgG reactive with thrombin [37] biotinylated using a DSB-X biotin labelling kit (Invitrogen, La Jolla, CA) and developed using a Qdot 625-streptavadin conjugate (Invitrogen) visualized at 302 nm with a GelDoc XR imaging system (BioRad, Mississauga, ON).

### Screening of phage display libraries by biopanning

T7Select10-3b API (P2-P1ran) or T7Select10-3b API (P7-P3ran) bacteriophage in titered liquid lysates were diluted to 10^9^ pfu/ml in 3% (w/vol) bovine serum albumin (BSA) in PBS in the presence or absence of 0.5 nM thrombin for 30 minutes at 37°C (total volume 1.0 ml). Thrombin was inactivated by addition of phenylalanyl-L-prolyl-arginine chloromethylketone (Calbiochem, La Jolla, CA) and phages were reacted with biotinylated anti-prothrombin IgG for 30 minutes at ambient temperature, with constant rotating. This incubation was repeated after addition of 0.075 ml of streptavidin-coated magnetic beads (FlowComp Dynabeads, Invitrogen). Beads were concentrated at the bottom of the tube using a magnet and washed ten times, for five minutes each, in 1.0 M NaCl, 1% (v/vol) Triton X-100 in PBS. They were then magnetically concentrated, resuspended in 0.5 ml PBS, and added directly into a culture of *E. coli* BLT5615 that had been grown to OD_600_ of 0.5 at 37°C in LB supplemented with 0.1 mg/ml sodium ampicillin, and induced with 1.0 mM isopropylthiogalactoside for 30 minutes. The infected culture was incubated at 37°C with shaking until lysis took place (1–3 hours), when it was clarified by centrifugation for 10 minutes at 8,000 X g. The phage lysate was refrigerated and used to re-start the procedure in additional rounds of biopanning. A total of five rounds of screening of each library was performed, with or without thrombin. The procedure is illustrated schematically in Figure 1B. After five rounds, the thrombin-biopanned and the mock-biopanned libraries were separately plated, and well-isolated plaques were scraped with a sterile pipette tip, adherent phages were eluted in 65°C water for 15 minutes, and one fifth of the eluent was PCR-amplified as described above, using primers 5918 and 5919. The purified PCR products were then directly DNA sequenced using primer 5919 at the MOBIX Central Facility, McMaster University.

### Probing of plaque lifts with anti-API antibodies

In some experiments, plaques formed on the surface of *E. coli* BLT5615/agarose/agar 100 mm Petri plates were transferred to circular discs of nitrocellulose paper and probed with horseradish peroxidase-conjugated sheep anti-human API antibodies (Affinity Biologicals) using a protocol analogous to that previously employed for immunoblotting of SDS-PAGE [37].

### Transfer of phage display sequences to a plasmid expression system

Approximately 1–2×10^8^ pfu of phages biopanned for five rounds with or without thrombin were PCR-amplified exactly as described above for DNA sequencing, but was then digested with PmlI and SauI and the 458 bp double digestion product was inserted between these sites in phosphatase-treated pBAD-H_6_API M358R [29]. Following ligation and transformation of *E. coli* TOP10 (Invitrogen) to ampicillin resistance, individual ampicillin-resistant colonies were picked from LB/ampicillin agar plates, scraped on a gridded archive plate, and used to inoculate 6.0 ml LB/ampicillin cultures. After overnight growth at 37°C with rotary shaking, arabinose was added to 0.002% (vol/vol) to induce recombinant API expression. Microcentrifuged pellets were lysed by sonication in PBS using a Sonic Dismembrator model 100 sonicator (Fisher Scientific). The ability of API-related proteins in the clarified bacterial lysate to bind immobilized thrombin was then measured in a microtiter plate assay, described below. Clones of interest were further investigated by preparing plasmid DNA mini-preparations from cultures inoculated from the gridded archive plate using a Pure Link Quick Plasmid Miniprep kit (Invitrogen). Plasmids were then DNA sequenced using primer 5919 at the MOBIX Central Facility, McMaster University.

Two plasmids expressing API P7–P3 variants DLTVS and LATVS, which represented consensus sequences from thrombin-panned phage display and the subsequent bacterial expression screen, were specifically constructed, since they were not found in the screening experiments. PCR using primers 4980 and antisense primers 2841 (5′-TGACCTCAGG CGGGATAGAT CTGGGAGACA CGGTCGCCAG CATGGCCCCA GCAGCTTC-3′) and 2842 (5′-TGACCTCAGG CGGGATAGAT CTGGGAGACA CGGTCAGATC CATGGCCCCA GCAGCTTC-3′) was employed, followed by restriction with PmlI and SauI and insertion between these sites in phosphatase-treated pBAD-H_6_API M358R.

### Thrombin capture assay

Purified thrombin (5 µg/ml) (Enzyme Research Laboratories) was coated onto each well of an Immulon 4 HBX 96 well microtiter plate (Thermo, Milford, MA) in PBS overnight at 4°C. All solutions applied to wells were 0.1 ml in volume. Wells were washed with 0.1% Tween 20 in PBS (PBST) and blocked using 5% (w/vol) non-fat skim milk powder in PBST for one hour at ambient temperature. Wells were then washed with PBST and the cleared bacterial lysates were applied neat (in duplicate) and allowed to react for one hour as described [38]. PBST washes were repeated and wells were next probed with 0.0002 mg/ml horseradish peroxidase- (HRP-) conjugated, affinity-purified sheep anti-human API (Affinity Biologicals, Ancaster, ON) for one hour. After final washes with PBST, HRP substrate 3, 3′, 5, 5′ - tetramethylbenzidine (TMB; Thermo Scientific, Rockford, IL) was applied and colour was allowed to develop for 15 to 30 minutes. The reaction was then stopped by application of 0.1 ml 2M sulphuric acid and the plate was read at 450 nm using an ELx808 plate reader (BioTek, Winooski, VT). Optical densities of samples to which buffer, rather than lysate, was applied were taken as background, and the mean of triplicates of such controls was subtracted from all lysate-containing readings.

### Kinetic characterization of API M358R and other variants

Recombinant hexahistidine-tagged API proteins expressed by *E. coli* TOP10 cells transformed with pBAD-H_6_-API plasmids were purified to homogeneity using nickel chelate affinity and ion exchange chromatography as previously described [29], [39]. The rate of reaction of recombinant API proteins was quantified by determining the second order rate constant (k_2_) of thrombin inhibition in a discontinuous assay, under pseudo first order conditions, as in previous studies from this laboratory [29], [40], [41], as was the stoichiometry of inhibition (SI). The SI is the number of serpin molecules required to inhibit one protease molecule, and was determined as previously described, by reacting various ratios of thrombin with API proteins, plotting the ratio versus the residual thrombin activity, and using linear regression to extrapolate to zero [29], [40], [41].

### Deep sequencing

T7 phage library DNA was extracted with phenol/chloroform, ethanol precipitated, and resuspended in 200 µL of 100 mM Tris-Cl pH 8.0 buffer. T7 library DNA (50 ng) from each experiment was subjected to PCR amplification with primers flanking the P7–P3 variable region. The primer design was similar to previously published sequences [42] but the adapters were re-designed to be compatible with Ion Torrent (Life Technologies, Guilford, CT) sequencing (See Figure S1 a scheme of primer design and Table S1 for a list of barcoded primers). The library from each experiment was amplified using five separate barcoded primers to test for any variability during PCR and sequencing. The PCR fragments were pooled together and gel-purified to isolate the band corresponding to the expected double-stranded DNA product. The concentration of DNA was determined using a Qubit Fluorimeter (Invitrogen), following the manufacturer's protocol. Ion Torrent protocols were used to ligate the DNA fragments onto Ion Sphere Particles (ISPs), amplify them by emulsion PCR, enrich the templated ISPs, and load onto an Ion Torrent 316 chip. FASTQ data files were processed using custom MatLab scripts that identified the barcodes, adapters, and the reads of the correct length and structure (See Figure S1A–C). Five sequencing runs of the same biological sample yielded reproducible copy numbers (See Figure S1D). We used goodness of fit statistics, as described previously [43] to show that sequence copy numbers in the replica runs were normally distributed (Figure S1E–F). This normality justified the use of parametric statistics (two sides, unequal variance t-test) to calculate the significance of the difference between the libraries in volcano-plots. We note that p-values obtained in this manner were based on technical replica (repeated preparation and sequencing of the same library). The scripts used to generate Scheme 6D–F and S1 are available as (Data S1, Data S2, Data S3).

### Computer software

DNA sequences were analysed with Clone Manager 7.11 and Align Plus 5.11 (Sci-Ed Software, Cary, NC). Amino acid sequences alignments used ClustalW 2.0. Amino acid conservation was quantified with ConSurf. API crystal structure image was produced using PyMol 1.3 release 1 (Schrodinger LLC, Cambridge, MA). Statistical analysis not related to deep sequencing was performed using GraphPad InStat version 3.06 (GraphPad Software, San Diego, CA).

## Results

### API M358R fused to T7 phage capsid protein 10B forms complexes with thrombin

**Figure 2:**
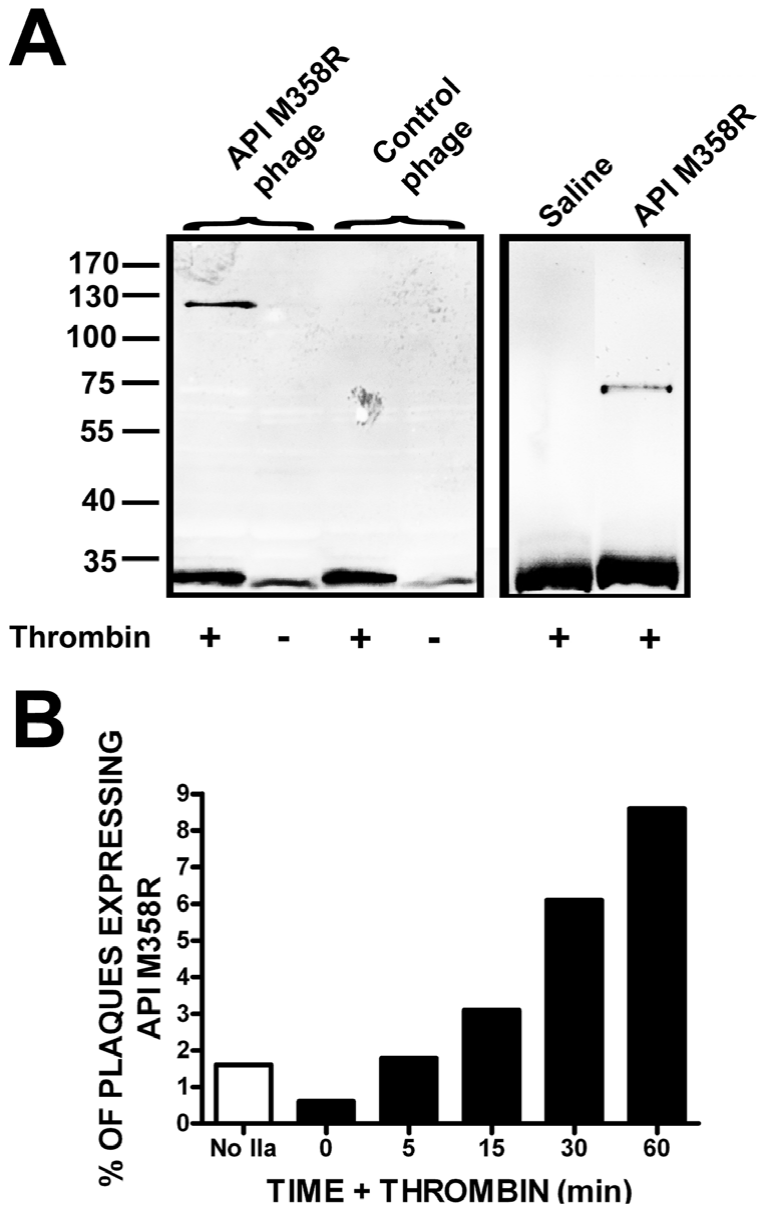
Reactivity of T7 10B-API M358R fusion proteins displayed on intact phage with thrombin and time course of enrichment of API M358R-expressing phage by exposure to thrombin. Panel A: 1×10^10^ pfu of purified T7Select10-3b API M358R (API M358R phages) and T7Select10-3b S-tag phages (Control phage) were separately reacted with (+) or without (−) 20 nM thrombin. Reactions were solubilized with SDS, electrophoresed on SDS-polyacrylamide gels under reducing conditions, immunoblotted, and probed with an affinity-purified sheep anti-thrombin antibody. Molecular weight marker positions, in kDa, are indicated to the left of the figure. The left panel represents four contiguous lanes of a single immunoblot. The right panel shows the reaction of 20 nM thrombin with 8.5 nM purified, soluble API M358R or saline controls; both lanes were derived from the same immunoblot but were not contiguous on the original image. Panel B: T7Select10-3b API M358R phages and T7Select10-3b S-tag phages were combined 1:100 and reacted with 0.5 nM thrombin for times shown on the x axis, prior to biopanning with biotinylated anti-thrombin IgG and streptavidin-linked magnetic beads. Washed beads were used to infect E. coli and aliquots of the resulting lysates used to form plaques on agarose/agar plates. Nitrocellulose plaque lifts were probed with anti-API antibodies to determine the percentage of immunoreactive plaques as a fraction of the total, shown on the y axis. “No IIa” (open bar) refers to a sample from the original mixture of phages subjected to plaque assay directly, without biopanning.

We previously demonstrated that API M358R retained the ability to form denaturation-resistant complexes with thrombin when fused to the C-terminus of non-cleavable signal sequence domains from either the human asialoglycoprotein receptor or the human transferrin receptor, ranging from 66 – 95 amino acids [37]. In the T7Select phage display system, proteins of interest for phage display are fused to the C-terminus of a considerably larger protein, the 348 amino acid T7 capsid protein 10B. Our first priority in this study was therefore to determine if API M358R retained thrombin inhibitory function when fused to the C-terminus of T7 10B. Purified phages were incubated with thrombin and the total reaction mixture was characterized by immunoblotting with an antibody specific to thrombin. As shown in Fig. 2A (left panel), SDS-stable complexes immunoreactive with the thrombin antibody were detected in reactions containing phages expressing the 10B-API M358R fusion protein but not in those containing phages expressing 10B fused to an unrelated S tag peptide. Unreacted thrombin was also visualized, as the 34 kDa B chain. A faintly reactive polypeptide, migrating slightly more rapidly than the thrombin B chain, was also observed; its presence in lanes containing only control phage suggested that it was a phage protein demonstrating cross-reactivity with the anti-thrombin antibody under the conditions employed. The approximately 110 kDa mobility of the denaturation-resistant serpin-thrombin complex was consistent with that expected of a fusion protein comprised of 348 residues of 10B, a Ser residue introduced in cloning steps, residues 1-358 of API M358R (after cleavage at R358-S359), connected via an acyl linkage to the B chain of thrombin (259 residues). A similar complex, of more rapid mobility, was observed in parallel reactions involving soluble recombinant API M358R and thrombin (Fig. 2A, right panel).

In order to demonstrate denaturation-resistant complex formation (Figure 2A) it was necessary to use higher phage titers and thrombin concentrations than were practical for phage display biopanning. To establish an appropriate reaction time under actual biopanning conditions, we mixed T7Select10-3b API M358R phages with T7Select10-3b S-tag phages at a 1 to 100 ratio, and incubated 10^9^ phages with 0.5 nM thrombin for varying times. Following selection with biotinylated anti-thrombin antibodies and streptavidin-coated magnetic beads, E. coli hosts were directly infected with biopanned bead-absorbed phage and plaque lifts probed with anti-API antibodies were used to assess the proportion of phage expressing API M358R. As shown in Figure 2B, in the naïve, unselected mixture, (“No IIa” column) only 1.6% of plaques expressed API M358R; the proportion was essentially unaltered by selection with thrombin for 5 minutes, but steadily increased with longer incubations of 15–60 minutes. On the basis of this preliminary experiment, we fixed the time of selection with thrombin at 30 minutes.

### Biopanning the T7Select10-3b API (P2-P1ran) phage display library

**Figure 3:**
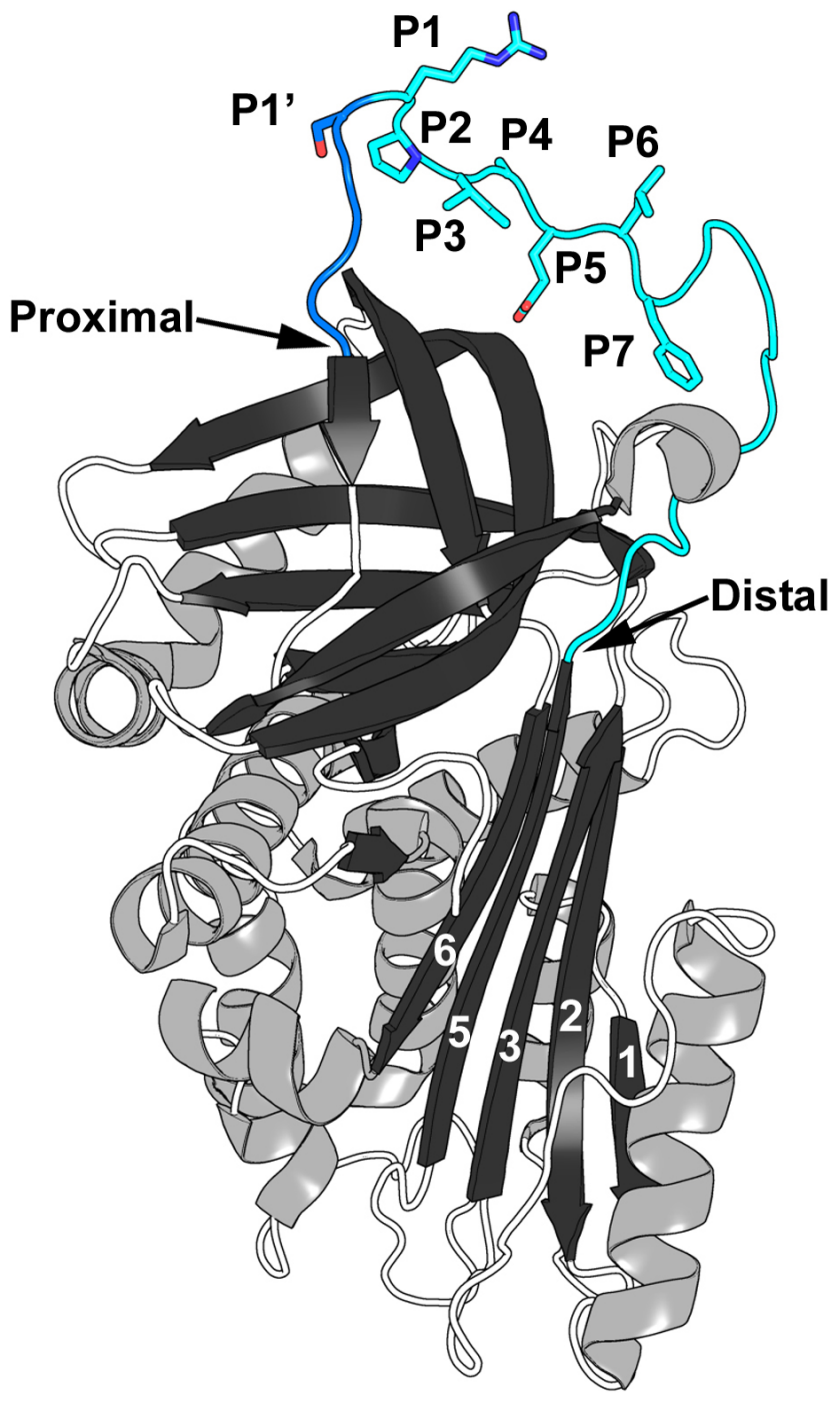
The structure of API M358R. PDB file 1OPH [49] was manipulated in PyMOL [59] to emphasize the RCL. β-sheets are shown in black and α-helices in gray, with random coiled segments of the polypeptide chain as open (white) coils. The β-strands of β-sheet A are numbered, in white. Note that the reactive centre loop (RCL) inserts between β-strands 3 and 5 as new strand 4 on complex formation with a cognate proteinase. The RCL is shown in blue, with the P (distal) side in light blue, and the P′ (proximal) side in dark blue. RCL residues between P7 and P1′ are shown as sticks to highlight their side chains and numbered in the image; the Proximal and Distal ends of the RCL are highlighted by arrows.

**Figure 4:**
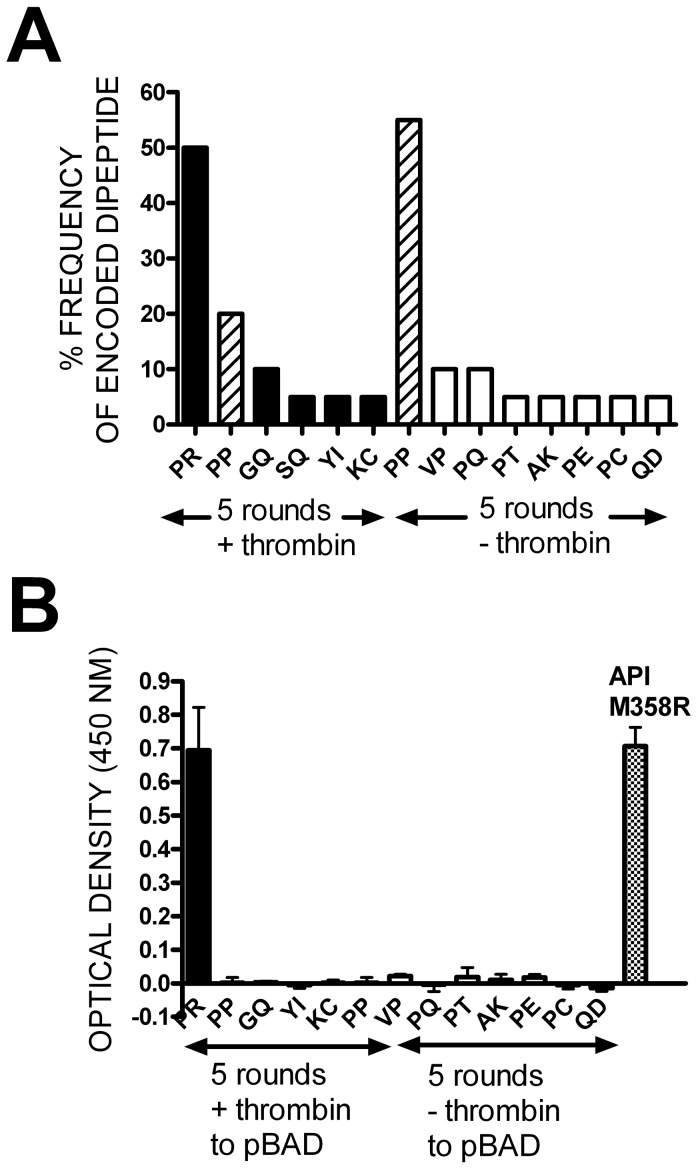
Characterization of API P2-P1 variants identified by sequencing of plaque-purified thrombin-selected or mock-selected phages. After biopanning the P2–P1 phage display library for five rounds with (+) or without (−) thrombin, the API inserts from 20 plaque-purified phage were sequenced from each biopanned library. Panel A shows the observed frequency, as a percentage, for variants with the dipeptide sequences shown on the x axis. Black bars correspond to variants identified by thrombin panning, while white bars correspond to those identified by mock selection; hatched bars identify the PP sequence identified in both groups. Panel B shows API variants with the same P2–P1 dipeptide sequences as in Panel A, but transferred to plasmids (pBAD) for expression as soluble, intracellular proteins. Cultures containing plasmids expressing the API P2-P1 dipeptide identified on the x axis were lysed and the extent to which anti-API immunoreactive proteins bound to thrombin immobilized on microtiter plate wells was quantified as the optical density at 450 nm. The mean of duplicate determinations ± SD is shown. As in Panel A, black bars relate to thrombin-panned and white bars to mock-selected candidates. Cross-hatched bar shows the results from a colony transformed with pBAD-H_6_API M358R as a positive control.

We further established conditions for biopanning phage display libraries with thrombin by mixing T7Select10-3b API M358R phages with T7Select10-3b S-tag phages at a 1 to 300 ratio and conducted five rounds of selection to model the screening of a hypervariable library. Using stringent high salt/detergent washes and direct infection of E. coli hosts with phages bound to streptavidin-coated magnetic beads via biotinylated anti-thrombin antibodies, we found that the proportion of phages expressing API M358R rose from <1% in the naïve initial “library” to >40% after five rounds of selection (data not shown). We therefore applied this protocol to a true library. We constructed a T7 phage display library expressing API randomized at codons 357 and 358 (P2–P1) (Figure 3 illustrates the RCL residues most relevant to this study). As a quick sampling, we sequenced the RCL of five plaque-purified phages from the naïve library, finding that all contained different P2–P1 codons and no other alterations in the rest of the RCL; the absence of P361 CCC codons also indicated no carryover. We then applied the protocol validated in the 1∶300 experiment described above, biopanning the library for five rounds with or without thrombin. As shown in Figure 4A, sequencing of the API RCL codons encoded by 20 individual plaque-purified phages selected with thrombin over five rounds revealed that 50% contained P357/R358 (PR), 20% had PP codons at the randomized positions, and four unique dipeptide sequences were present at 10% or less in the rest of the group. Out of 20 individual plaque sequences from the mock-selected group not exposed to thrombin in its five rounds of panning, 55% had PP codons at the randomized positions, and seven unique dipeptide sequences were present at 10% or less in the rest of the group. Only phages with PP at P2–P1 were present in both groups.

To gain insights into the functionality of the selected variants, we transferred the P2P1 variant RCL sequences into the pBAD-H_6_API M358R plasmid for arabinose-inducible expression of soluble, intracellular, hexahistidine-tagged API P2-P1 variant proteins. When E. coli TOP10 cells harbouring pBAD-H_6_API M358R were lysed, API M358R bound to thrombin immobilized on microtiter plate wells and was readily detected by specific anti-API antibodies conjugated to HRP, as indicated by strong colour generation in the presence of chromogenic substrate (see Figure 4B, extreme right panel). When lysates containing the P2P1 variants identified in the phage display screen were tested in the same assay, only PR produced a strong signal; all other dipeptide candidates reacted at background levels.

### Biopanning the T7Select10-3b API (P7-P3ran) phage display library

Having demonstrated the utility of biopanning T7 phage display libraries to select functional API variants, we sought to apply the technique to a larger segment of the RCL without altering the protocol employed for the P2–P1 library. We allowed all four nucleotides at each position of 5 contiguous RCL codons, a total of 4^15^possible different DNA sequences, or approximately 1×10^9^ possibilities, the same number of phage screened per round in our protocol. Because 64 codons encode 21 amino acid or stop codons, the number of possible different displayed protein sequences would tend to be, on average, 3-fold lower than the DNA diversity. Whether or not we could PCR-amplify all possible DNA sequences, or, for that matter, package all recombinant phage DNA molecules, was unknown; however, these estimates made it clear that there was little point in seeking to randomize more than 5 residues at a time. Accordingly, we next constructed a T7 phage library displaying API randomized at codons 352- 356 (P7–P3) (see Fig. 2). A small sampling of ten plaque-purified phages from the naïve API P7–P3ran library revealed different pentapeptide codons in the appropriate positions, no alterations outside the intended hypervariable region, and no carryover of API M358R from other DNA sources. The titer of the naïve library, prior to biological amplification, was approximately 4×10^6^.

Following five rounds of biopanning with or without thrombin, the hypervariable codon sequences of forty plaque-purified phages from each group were determined. As shown in Table 1, six pentapeptide sequences were found more than once in the thrombin-panned population, as well as thirty-three unique sequences. Alignment of the pentapeptides using ClustalW software revealed a consensus sequence of DLTVS (compared to the native FLEAI sequence), based upon the frequency of each amino acid in the 40 sequences. In contrast, the mock-selected library was considerably less diverse; three pentapeptide sequences were present in 65% of the plaque-purified phages (PPAPL, FGSNI, and RTFIN), with the remainder made up of only seven different sequences; no discernible consensus sequence was found.

**Table 1:** Frequency of Specific P7–P3 sequences in biopanned phage display libraries.

Source	P7–P3 Sequence&	Frequency (%)#
Wild type	FLEIA	0
Round 5 thrombin-panned plaques	NLIPT	7.5
	DITMA	5
	DAFVT	5
	QPPPS	5
	PLFVS	5
	SLELK	5
	DAFAA, TAHVT, QATFL, EAHFR, HPPSL, *PKSEG*, DATVS, DVTVS, HATIS, HATVS, YATLS, TLSAV, LVFVS, PLQLS, EASLI, SLAMT, ELLAA, ELLAL, QLTAT, LLPYS, PLAPI, QRPHQ, RYHYI, PAMPR, ARSST, WNPVI, NFCAI	2.5 (each)
Round 5 mock-panned plaques	PPAPL	32.5
	FGSNI	30
	RTFIN	12.5
	*PKSEG*	5
	LTMTN	5
	PFMLH	5
	IGHTA, VPVVI, SHRLP, RLLVK,	2.5 (each)

^#^ The RCL within API M358R cDNA sequences present in forty plaques plated after 5 rounds of biopanning with (thrombin-panned) or without (mock-panned) thrombin was sequenced and the frequency expressed as the number of times a given sequence was found, as a percentage.

^&^ Italicized sequence was found in both round 5 thrombin-panned and round 5 mock-panned plaques.

### Soluble expression screening of the thrombin-selected phage sequences in bacteria

**Figure 5:**
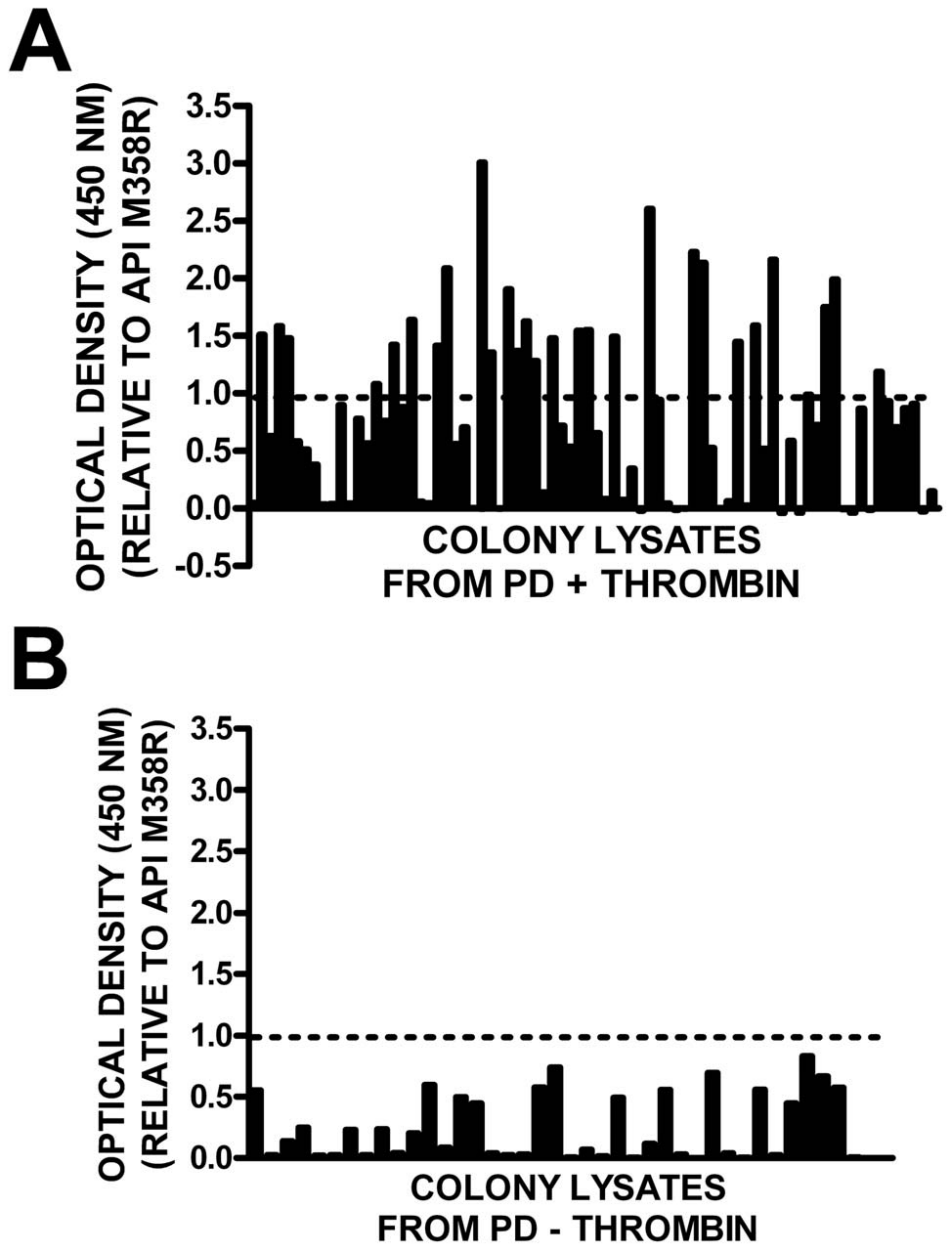
Relative binding of API P7–P3 variants in bacterial lysates. Panel A: Thrombin-selected API RCL inserts from round 5 of phage display were transferred en masse to a plasmid expression library and bacteria were transformed. Eighty colonies were screened by the thrombin capture assay (as shown in Fig. 4B) and the resulting optical density values were normalized to API M358R lysate controls. The mean of two determinations is shown. Panel B: Same as panel A, except that the source of the plasmid expression library was round 5 of mock selection. The horizontal line in both panels highlights the relative optical density ratio of API M358R in the normalized results (y = 1.0).

In view of the considerable diversity of the quintuply-selected thrombin-reactive P7–P3 hypervariable sequences, we sought an additional means to distinguish faster-acting from slower-acting variants with respect to thrombin inhibition. Accordingly, we PCR-amplified the round 5 thrombin-selected phage RCL sequences en masse and transferred them into pBAD-H_6_API M358R. Transformation of E. coli TOP10 with this mass population produced a pre-selected API M358R API (P7–P3ran) bacterial expression library. Lysates were prepared from eighty randomly selected colonies from this library, and assessed in the thrombin capture assay (previously shown in Fig. 4B). As shown in Figure 5A, 27 of 80 colonies exhibited optical density values in the assay greater than API M358R. In contrast, none of 40 bacterial clones containing RCL sequences from the quintuply mock-selected phage library exhibited thrombin binding to a greater extent than API M358R (Figure 5B). As shown in Table 2, sequencing of plasmid DNA preparations from the supra-reactive thrombin-binding candidates revealed that 22 of the supra-reactive candidates were unique sequences; Clustal W profiling revealed a consensus sequence of LATVS, while ConSurf software analysis generated a loose consensus of P7-Not Aromatic/P6-Hydrophobic/P5-T or S/P4-Hydrophobic/P3-Not Aromatic.

**Table 2:** P7–P3 variants binding thrombin in a capture assay more effectively than API M358R.

P7–P3 Sequence	Frequency (%)&	Relative OD_450_ versus API M358R^#^
AAFVS	3.7	3.01
EISLQ	3.7	2.61
FHTLG	3.7	2.13
LTTLR	3.7	2.09
HATVS	3.7	1.99
QATFL	7.4	1.91
VTTIT	3.7	1.90
LASMR	7.4	1.89
DITMA	3.7	1.64
DAFAA	3.7	1.59
EATVS	11.1	1.54
LINPI	3.7	1.54
EAHFR	3.7	1.51
TVSVS	3.7	1.49
ISSAN	7.4	1.46
LAITS	3.7	1.45
DVTVS	3.7	1.42
QVKPA	3.7	1.37
TLSAV	3.7	1.36
LSELA	3.7	1.28
LGSFT	3.7	1.19
RVNAK	3.7	1.08

^&^ The RCL within API M358R cDNA sequences present in 27 bacterial clones with higher thrombin capture assay values than API M358R was sequenced and the frequency expressed as the number of times a given sequence was found, as a percentage.^#^The mean of two determinations on one lysate is shown for all variants present at 3.7% frequency, of two lysates for 7.4% frequency, and of three lysates for 11.1% frequency.

A number of candidate sequences were found in both plaque and lysate screens (DAFAA, DATFL, DVTVS, DITMA, QATFL, HATVS, and TLSAV). Prior to selecting candidates for purification and kinetic analysis, we specifically transferred the RCLs bearing the pentapeptide sequences that were detected more than once in the plaque-purified sequencing experiments (NLIPT, PLFVS, DAFVT, SLELK, and QPPPS). As shown in Table 3, rightmost column, of these, only NLIPT gave a lysate assay optical density value greater than API M358R. Finally, we also made plasmid constructs specifically expressing the plaque-purified or lysate-screened consensus sequences, respectively (DLTVS and LATVS) which yielded lysate values slightly higher than API M358R. Twelve P7–P3 variants were then selected for analysis on the basis of the strength of their thrombin binding activity in the lysate assay or their abundance in the array of plaque-purified sequences or their representing consensus sequences (see Table 3).

**Table 3:** Kinetic properties of API M358R variants with P7–P3 substitutions.

P7–P3 Sequence	Selected by#	k_2_ (x 10^6^ mol^−1^ sec^−1^)	SI	Relative OD_450_ versus API M358R (lysate)
FLEAI	Wild type	0.487±0.0427	2.00±0.03	1.0
AAFVS	Lysate	1.01±0.073*	1.74±0.17	3.0
DITMA	Plaque, Lysate	1.00±0.0691*	1.57±0.11	1.7
LTTLR	Lysate	0.896±0.145	2.63±0.26	2.1
LASMR	Lysate	0.895±.0871	2.03±0.08	1.9
LHTLG	Lysate	0.786±0.126	2.01±0.16	2.1
EISLQ	Lysate	0.783±0.0934	1.97±0.17	2.6
LATVS	Lysate consensus	0.765±0.129	2.01±0.10	1.5
EATVS	Lysate	0.617±0.0517	1.86±0.12	1.5
QATFL	Plaque, Lysate	0.593±0.141	2.04±0.20	1.9
HATVS	Plaque, Lysate	0.504±0.0848	2.67±0.16	2.0
DLTVS	Plaque consensus	0.529±0.0685	1.85±0.13	1.1
NLIPT	Plaque	0.176±0.0313	5.8±0.6	1.3
PLFVS	Plaque	ND	ND	0.83
DAFVT	Plaque	ND	ND	0.63
SLELK	Plaque	ND	ND	0.63
QPPPS	Plaque	ND	ND	0.03

p<0.05 by Kruskal Wallis non-parametric ANOVA, with Dunn's post-tests, relative to wild type k_2_; k_2_ and SI values are the mean ± SD of 5 determinations.

^#^ The following terms were used to explain how sequences were selected for inclusion in this table: “Lysate” indicates selection by high value in thrombin capture assay of bacterial lysates of cells expressing the indicated API M358R variant; “Plaque” indicates selection by presence in the group of 40 plaques whose API M358R RCL was determined by PCR and sequencing after 5 rounds of biopanning with thrombin; “Plaque consensus” indicates the most common residue at P7–P3 from the 40 plaque sequences; “Lysate consensus” indicates the most common residue at P7–P3 from the 22/80 lysates from colonies in the bacterial lysate thrombin capture assay screen shown in Fig. 5.

### Kinetic analysis of purified API M358R P7–P3 variants

Twelve novel API M358R proteins varying between P7–P3 were purified and their thrombin inhibition rate constants were determined. As shown in Table 3, mean rates of thrombin inhibition were greater than that of API M358R in all cases, with the exception of the NLIPT variant, which exhibited a rate constant 2.8-fold lower than API M358R. P7–P3 variants AAFVS and DITMA were found to have the greatest increase over API M358R, of 2.1-fold in both cases; unlike all the other increased rate constants that had been observed, these increases were found to be statistically significant by ANOVA with post-tests of the entire group. Stoichiometries of inhibition (SI) were also determined. Most variants differed little from API M358R made in this system, although AAFVS and DITMA exhibited decreased SI values indicative of enhanced inhibitor activity and decreased substrate activity, and NLIPT had an elevated mean SI of 5.8, indicative of the opposite tendency. None of the alterations in SI reached the level of statistical significance.

**Figure 6:**
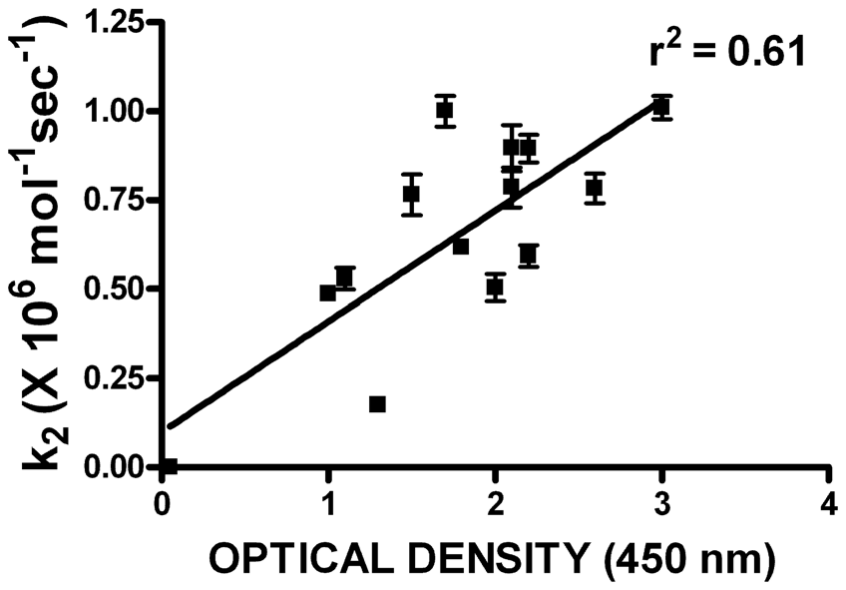
Relationship between kinetic rate constant and optical density in thrombin capture assay. The second order rate constant (k_2_; mean of 5 determinations ± SD) was plotted as a function of the optical density at 450 nm in the thrombin capture assay (mean of 2–3 determinations) for API M358R and the 12 P7–P3 variants with kinetic results shown in Table 3. The mean OD_450_ (n = 5) for mock thrombin capture assays lacking API-related proteins in the lysate (0.05) was taken as indicating a K_2_ of 0. The correlation of determination (r^2^) is shown next to the regression line.

Plotting the mean k_2_ values versus the mean optical density values in the bacterial lysate assay revealed a general positive correlation; the higher the optical density, the higher the k_2_, but the relationship demonstrated only moderate linearity (r^2^ = 0.61 by linear regression, as shown in Figure 6.

### Deep sequencing

Deep sequencing using the Ion Torrent platform was next applied to gain a better understanding of library sequence diversity, and to test the premise that sequence enrichment correlated with the use of the bait protein, thrombin (see Figure S1A for primer design). Quality control was applied to the data such that only reads of the correct length and containing correct sequences flanking the variable region codons were analyzed (Figures S1B and S1C). The naïve T7 API (P7–P3ran), the quintuply thrombin-panned library, and the mock-panned library were each subjected to deep sequencing. The results in each case were highly reproducible between reads (Fig. S1D). Deviation from the average copy number of the populations was observed to follow a noisy Poisson distribution sufficiently closely that parametric statistics could be employed to evaluate enrichment (Figure S1F).

**Figure 7:**
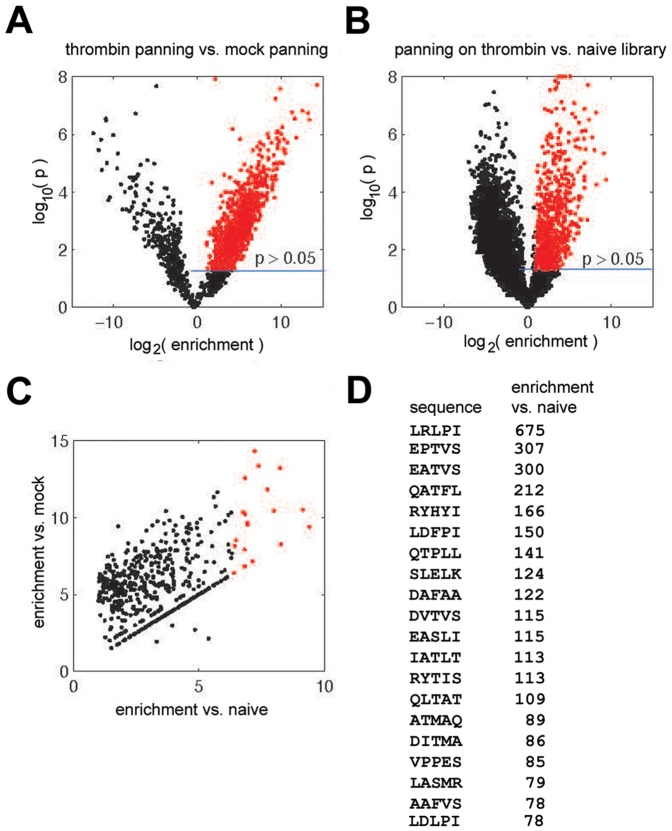
Panel A: Volcano plot showing enrichment of sequences in thrombin panning versus mock panning. Panel B: Enrichment of sequences with respect to naïve library. In (A) and (B), red dots describe sequences that have been enriched by factor >2 with significance level of p>0.05 (calculated by two-tail unequal variance t-test). Panel C: Plot of sequences that have been significantly enriched over two controls. Red dots represent the top 20 sequences that show the highest level of enrichment with respect to naïve library (ca. factor of 100 or more). The pentapeptide sequence of the top 20 most enriched sequences lacking stop codons is shown in Panel D, in order of enrichment. The sequence of all significantly enriched sequences (784 in total) is shown in Table S3.

Analysis of the deep sequencing data (Figure 7) confirmed that sequences were significantly enriched in the thrombin-panned library as compared to either the mock-panned or naïve libraries. The fold-enrichment versus significance level scatter plots (volcano plots, Figure 7A and 7B) showed two clear arms, with red dots comprising sequences enriched greater than 2-fold in the thrombin-panned library over the control library being found in the right arm exclusively, and forming the vast majority of members of this distribution with p>0.05. Figure 7C shows a plot of the enrichment of sequences in the thrombin-panned library versus both the mock-panned and naïve library, with the top 20 sequences (listed in Figure 7D) identified in red (upper right). Enrichment ranged from 78- to 676-fold in this group over the naïve library.

Of the twenty sequences, eight (DITMA, SLELK, DAFAA, QATFL, DVTVS, EASLI, QLTAT, and RHYHI) also appeared in the group of 40 plaque-purified thrombin-panned phages we sequenced traditionally (Table 1); none was found in the mock-panned phage traditional sequencing group. With respect to variant sequences that we found by traditional sequencing, inspection of the full list of enriched sequences (see Table S3), showed that an additional 11 pentapeptide sequences were found to be enriched greater than 20-fold, by the specific fold margins indicated in the parentheses: PLQLS (72); HATVS (51); EAHFR (45); SLAMT (36); HATIS (34); DAFVT (30); TAHVT (28); ELLAA (24); PAMPR (24); NFCAI (24); and PLFVS (22). Pentapeptide sequences EATVS, LASMR, and AAFVS, found in the bacterial lysate screen, but not in the 40 plaque-purified thrombin-panned phages, were also found to be enriched (by 300-, 79-, and 78-fold, respectively). In contrast none of the sequences found by traditional sequencing in the quintuply mock-panned library were found to be enriched (see Table S3). Thus, 19 of 40 variants found by traditional sequencing of thrombin-selected plaques were confirmed to be enriched >20-fold by five rounds of selection with thrombin by deep sequencing. While deep sequencing cannot be used to count accurately the number of clones in any of the libraries, and is itself dependent on PCR steps, its reproducible detection of over 10,000 unique sequences meeting quality control criteria in the naïve library gave some indication that we had succeeded in creating a diverse initial array (see Tables S2 and S4).

## Discussion and Conclusions

In this study, we demonstrated the utility of phage display for selecting variants of API with the ability to inhibit thrombin. We first showed that denaturation-resistant complexes could be formed between thrombin and API M358R, when this serpin was displayed on the surface of T7 phages, fused to the C-terminus of T7 capsid protein 10B. This orientation may have contributed to the reactivity of the displayed API M358R, because it left the C-terminal region of the serpin, home to the RCL, free of steric hindrance arising from proximity to either the 10B protein or the phage surface. Both potential interfering factors would likely have been greater had the orientation been reversed. Positioning the serpin at the C-terminus of the capsid protein also ensured that thrombin, any thrombin-complexing API variant, and the phages containing the vector encoding the variant RCL would remain linked following complex formation, if API 359–394 diffused away following reactive centre bond cleavage. Had the orientation been reversed, thrombin-bound API 1-358 could have diffused away from the phages, eliminating the ability to deduce the sequence of its variant RCL.

It is curious that, of 36 human serpins, many of which have been very actively investigated in terms of structure and function, only PAI-1 has been previously shown to be functional in phage display, primarily in M13-based systems. It has been suggested that phage display in T7 phages may allow for appropriate folding of a wider range of fused foreign proteins than M13, since T7 is assembled in the cytoplasm and released by lysis, as opposed to M13, whose constituent proteins must traverse a secretory pathway prior to assembly in the periplasm and secretion [44]. If PAI-1 happened to be fortuitously amenable to M13 display, as opposed to other serpins, this may explain the previous paucity of serpin phage display reports. For instance, in the 1990s our laboratory obtained the pComb3/M13 phagemid system used for phage display of PAI-1, under a materials transfer agreement, but was unable to demonstrate functional expression of either antithrombin (AT) or heparin cofactor II (HCII) in the system.

Our first application of the T7 phage display of API was a relatively modest screen involving randomization of only the P2 and P1 residues. A priori, it was known that native API inhibits thrombin at a very modest rate described by a k_2_ of 4.8×10^1^ M^−1^ sec^−1^ [45], and that the M358R mutation elevates this rate by 5 orders of magnitude [46]. Unlike native serpins that inhibit thrombin, the API RCL was not selected by evolution for this purpose, and only came to light through a pathological “experiment of nature”. Biopanning of the API P2–P1 randomized phage display library with thrombin yielded several sequences, of which API M358R was the most abundant. Further characterization of the selected sequences revealed that only API M358R yielded a functional inhibitor when its P2P1 residues were recreated in soluble recombinant API. The result was unsurprising given thrombin's known preferences for substrates with Pro at P2 [47]. That other sequences were found in the population of phages selected by thrombin in five rounds of phage display reinforced the concept that phage display is a screening approach, and that sequences unrelated to the complexing of thrombin by the API motif of the 10B fusion protein could also be selected (e.g. P2–P1 PP) by multiple routes. These included binding to some component of the assembly of molecules used in biopanning (IgG, streptavidin) or to the magnetic beads, or through cross-reactivity between phage coat proteins and the anti-thrombin antibody used for selection (despite its being affinity-purified). It is also possible that, for unknown reasons, these constructs had growth advantages that explained their abundance in the absence of thrombin selection.

Thrombin biopanning of a more diverse library of phage-displayed API M358R randomized at P7–P3 was similarly successful in enriching the population with variant serpins capable of inhibiting thrombin. This deduction was supported by several lines of evidence. First, minimal overlap was found between sequences selected by biopanning with thrombin and mock-selection in the absence of the protease. Secondly, the thrombin-selected sequences followed some previously noted general features of functional serpins, such as the consensus for hydrophobic residues at P6 and P4 [1] and a general tolerance for alterations differing greatly from the wild-type sequences at odd numbered, but not even-numbered residues of P5 and P3 [48]. Thirdly, most of the candidates in the thrombin-selected phages bound thrombin when transferred en masse to bacteria and screened by thrombin capture of bacterial lysates. Finally, deep sequencing, which unlike our traditional sequencing of selected representatives of mock-panned and thrombin-panned libraries, was technologically sufficiently robust to sample thousands of unique sequences multiple times, quantitatively confirmed enrichment of ∼800 variant RCL candidates in the phage display library biopanned with thrombin through five rounds.

The demonstrated utility of phage display for enriching thrombin-reactive P7–P3 variants still left us with an abundance of candidates, one too large to express, purify, and characterize kinetically in a search for supra-active variants. In addition, the reasonable assumption that variants that were more effective thrombin inhibitors than other candidates would be more frequently encountered in the selected population was observed to be valid for the P2–P1 screening, but not for the P7–P3, at least with respect to the forty plaque-purified candidates we sequenced traditionally. This lack of correlation could have arisen for at least two reasons: that our biopanning protocol was insufficiently stringent to distinguish fast from slow thrombin inhibitors; and/or that the small number of candidates whose hypervariable codons we determined by sequencing was insufficient to reveal the true abundance of different P7–P3 variants in the selected populations. We addressed the first possibility by conducting a “sixth round” of screening, transferring the quintuply-selected thrombin-panned API library to bacterial plasmids, and screening lysates semi-quantitatively for enhanced thrombin binding. This measure confirmed that the thirty minutes that we allowed for thrombin-API variant fusion protein binding during biopanning was likely long enough to encompass a range of thrombin inhibitors, some faster, and some slower, than API M358R. Nevertheless, it allowed us to hone in on the eight novel P7–P3 variants listed in Table 3 which demonstrated elevated rates of thrombin inhibition compared to API M358R on kinetic analysis. The two most active variants exhibited a statistically significant, 2.1-fold elevation in the rate of thrombin inhibition, relative to API M358R.

Supra-active P7–P3 variants DITMA and AAFVS, the two most active variants arising from the combined phage display/bacterial expression screening approach were quite different from each other; DITMA fit the P7-Not Aromatic/P6-Hydrophobic/P5-T or S/P4-Hydrophobic/P3-Not Aromatic loose consensus defined by our sequencing of strongly reactive candidates from the lysate screening, but AAFVS fit at only four of five positions, due to its central aromatic residue. Previously it had been suggested that the P5 glutamic acid residue in native API, observed to form salt bridges with a basic pocket in the serpin body in a crystal structure [49], was important for optimal inhibition of elastase and trypsin [50], providing needed rigidity in the RCL. This property does not appear to be important for inhibition of thrombin, based on the demonstrated superiority of DITMA and AAFVS to the native FLEAI pentapeptide sequence. RCL flexibility may be more important for API-mediated inhibition of thrombin than the other proteinases. Smaller polar residues such as Thr and Ser at P3 may provide a better fit to thrombin's substrate site than Glu; in this regard, Met and Val may fill thrombin's known S4 hydrophobic pocket more effectively than the native, smaller Ala residue in the native sequence [47], [51], [52].

While many investigators have conducted mutagenesis studies of the API RCL, few have reported variants with increased rates of thrombin inhibition. Hopkins et al. reported a variant API M358R in which the P7–P3 residues of antithrombin (AVVIA) had been substituted for those of API, in addition to the I360L and P361N antithrombin- API exchange [53]. In the initial report, these investigators termed this protein “LS-Pro” and reported that its rate constant for thrombin inhibition was approximately 65% of that of API M358R. Subsequently, in an expanded study [54], Hopkins et al reported a rate constant for inhibition of thrombin 2.8-fold greater than API M358R by a closely related variant to LS-Pro lacking the P361N substitution (termed LS7-3/P′2L). In both reports, these investigators expressed a truncated form of API M358R in E. coli, one lacking its N-terminal 12 residues [53]. We expressed both API M358R and the LS7-3/P′2L variant, which we designated “API RCL5”, in a hexahistidine-tagged, full length recombinant API protein, finding, that it was less active than API M358R, in our hands by about 13% [30]. Thus, it is uncertain whether or not API variants with increased activity versus thrombin have been synthesized, prior to this study. It is however clear that DITMA and AAFVS inhibit thrombin significantly more rapidly than API M358R, when made in the same expression system and assessed kinetically at the same time.

We also assembled consensus variants DLTVS and LATVS, from the phage and bacterial sequencing experiments, respectively. Each was comprised of the most frequent amino acid found at each position. While the inhibitory rates of these variants were increased relative to API M358R, they were less active than DITMA and AAFVS. It would appear from this observation that cooperativity is active in the RCL, as has been previously suggested [54].

The enhanced reactivity of the DITMA and AAFVS variants, as well as most of the P7–P3 variants that we advanced to kinetic characterization, was accompanied by no increase in the stoichiometry of inhibition; SI values for these variants were slightly less than for API M358R. We have previously reported that API made in this soluble, intracellular bacterial expression system, exhibits SI values of 2–3[29], [30], [41]. Others employing different bacterial expression systems, some in which recombinant API was renatured from inclusion bodies, have reported SI values closer to unity [55], [56]. Why the difference arises is not fully understood, but we have demonstrated functionality of API M358R made in this system as an antithrombotic agent in vivo [39], suggesting that it does not have an unnatural fold. Deep sequencing verified that thrombin biopanning enriched many sequences over their abundance in either the naïve library or the mock-panned library, processed through the same five rounds of selection as the thrombin-selected population. The group of sequences most enriched by thrombin biopanning (in comparison to the naïve library) contained many candidates found by traditional sequencing of plaque-purified phage from five rounds of selection. Although not found at the level of enrichment of some candidates, DITMA and LASMR, shown to be more active than API M358R in the kinetic analysis, were also found to exhibit 86- and 79-fold enrichment, respectively. Using direct quantification of enrichment by deep sequencing in positive and negative panning experiments allowed us to bypass two previously observed unexpected outcomes in phage display screens: selection of fast-amplifying sequences (reviewed in [57]); and collapse of sequence diversity to a few hundred candidate sequences prior to biopanning [42]. Thus, the overlap between candidates found to be enriched by deep sequencing, found by traditional sequencing of plaques, and found by phage display overlaid with a semi-quantitative bacterial lysate expression screen, taken together, suggested the combined utility of all three approaches.

Like most screening approaches, phage display of API variants would likely be most effective in a situation in which the functional change conferred by hypervariable mutation was the greatest. This general principle was evidenced in the P2P1 screen, in which the >400 possibilities could be quickly narrowed down to API M358R. In contrast, while we succeeded in sifting through the many functional P7–P3 variants to find those with highest activity, considerably more effort was required. In retrospect, reducing the time that phages were exposed to thrombin during the biopanning process to 10–15, rather than 30 minutes, could have increased our yield of highly active P7–P3 variants. Using such further refined T7 phage display, complemented by a final round of bacterial expression screening, might pay the greatest dividends in future experiments that deliberately inactivated API, perhaps by creating an active centre not preferred by thrombin (e.g. Leu-Ser, the atypical thrombin cleavage site in HCII [58]) and seeking to rescue this inactivating mutation through compensatory changes elsewhere in the RCL. The approach could also be further refined by adding negative selection steps into the biopanning regimen, for instance with activated protein C, or substituting an entirely different protease and specific antibody combination for thrombin.

In conclusion, in this study we demonstrated that a second serpin retained functional activity when displayed on the surface of phage: API. We successfully adapted a T7 phage display system and used it to probe two hypervariable libraries, randomizing first two adjacent, and then five adjacent RCL positions. The results showed, in an unbiased and novel manner, that API M358R is optimal for thrombin inhibition at P2–P1. The P7–P3 phage display screen, complemented by bacterial lysate screening of a transferred, biopanned library, was effective in finding two novel variants that inhibited thrombin greater than twice as rapidly as API M358R. Deep sequencing was used to confirm that biopanning with thrombin significantly enriched numerous pentapeptide sequences over the naïve library, and also provided additional enriched candidates missed in traditional sequencing of a small subsample of the library. We conclude that phage display of API is a promising additional tool in the armamentarium of researchers interested both in structure/function studies of this archetypal serpin and in engineering it for altered reactivity and/or specificity.

## Supporting Information

Figure S1 **Panel A: Description of the primers used for Ion Torrent sequencing and the structure of the displayed on 10b-displayed insert surrounded by the primer sequences.** N is a random nucleotide. Panel B: Representative distribution of read length after Ion Torrent analysis. Panel C: Analysis of the reads of the wrong length revealed insertions/deletions and homopolymer read errors which are common in Ion Torrent reads [60], [61]. Only the reads that contained correct length and correct sequence flanking the (N)15 insert were considered (see Table S2). Panel D: We re-sequenced each library five times and observed that copy numbers were reproducible between the reads. The plots show deviation from an average copy number. Panel E: To show that deviation between sequencing runs follows a noisy Poisson distribution [62], we calculated goodness of fit statistics X(i) for each read i. Panel F: We compared the distribution of X to the Chi-squared distribution with 4 degrees of freedom using a QQ-plot [43]. The thrombin-panned and naïve libraries were found to be normally distributed. The ∼1.2 slope on the QQ-plot indicates that the variance of the reads is 20% higher than the variance of the Poisson distribution (with the increase being due to noise in PCR, emulsion PCR and re-sequencing). In mock panning, a small number of reads deviated from normal distribution; but the majority of the library was found to be normally distributed. As the copy numbers were normally distributed, we used parametric statistics (t-test) to evaluate the enrichment in the screen (see Figure 7). The complete processed deep sequencing data set, showing all (N)15 insert sequences found in this study, is also provided in Table S4 (see below).
(PDF)

Table S1 **List of Barcodes used in Ion Torrent primers.** The DNA sequence of the 18 different barcodes used in Ion Torrent oligodeoxyribonucleotide primers is listed, in standard 5′ to 3′ orientation.
(DOC)

Table S2 **Number of reads identified by Ion Torrent sequencing for different experiments.** On the 9 column by 5 row table, “+IIa” indicates selection with thrombin (IIa), “-IIa” selection without IIa, and “N” indicates the naive P7–P3 randomized library; r1, r2, and r3 refer to different deep sequencing replicas. Only three out of five replicas are shown here (but see additional Supporting Information listed below). In the rows, “Total” indicates the total number of reads identified for a specific barcode; “match” refers to the number of reads that match to PCR primers; N55 identifies the number of reads that have the correct insert length; N15 refers to the number of reads that reflect the correct structure of the library; and “Unique” corresponds to the number of unique sequences.
(DOC)

Table S3 **Sequences enriched in the API P7–P3 phage display library quintuply biopanned with thrombin (round 5), versus the naïve library.** The first column identifies the translated P7–P3 amino acid sequence, in single letter code (with asterisks indicating termination codons). The second column shows the fold enrichment over the naïve library. Note: the top 20 enriched sequences are also shown in Figure 7D.
(TXT)

Table S4 **Complete deep sequencing data set from this study.** The table contains 12626 DNA sequences and 5 replicas of sequencing for positive and mock selection, and also 5 replicas of sequencing of the naive library.
(TXT)

Data S1 **A MatLab script that generates Figure S1 from the Table S4 file (for PC operating systems). Scripts are based on a published MatLab data analysis suite** [**63**] **.**
(M) Click here for additional data file.

Data S2 **A satellite MatLab script used by the Data S1 file (for PC operating systems). It must be present in the same folder for the Data S1 MatLab script to work.**
(M) Click here for additional data file.

Data S3 **A MatLab script that generates** **Figure 7** **from the Table S4 file (for PC operating systems). It also generates the Table S3 file.**
(M) Click here for additional data file.
