# Correction: Phage Display of the Serpin Alpha-1 Proteinase Inhibitor Randomized at Consecutive Residues in the Reactive Centre Loop and Biopanned with or without Thrombin

**DOI:** 10.1371/journal.pone.0238969

**Published:** 2020-09-10

**Authors:** Benjamin M. Scott, Wadim L. Matochko, Richard F. Gierczak, Varsha Bhakta, Ratmir Derda, William P. Sheffield

In Fig 3, the labels for Proximal and Distal are swapped. The right-hand label should be Proximal and the left-hand label should be Distal. Please see the correct Fig 3 here.

There are errors in the caption for Fig 3, “The structure of API M358R. PDB file 1OPH [49] was manipulated in PyMOL [59] to emphasize the RCL.” Please see the complete, correct Fig 3 caption here.

**Fig 3:**
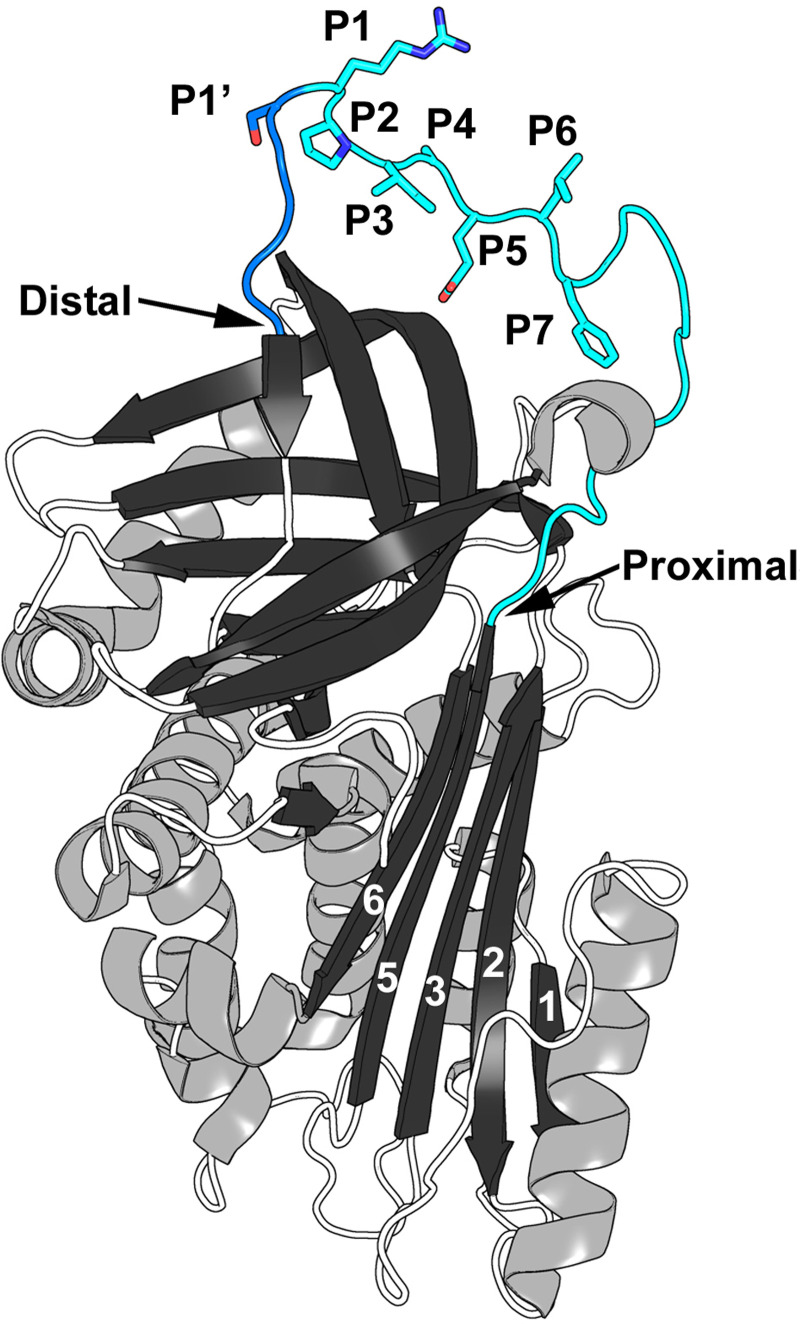
The structure of API M358R. PDB file 1OPH [49] was manipulated in PyMOL [59] to emphasize the RCL. β-sheets are shown in black and α-helices in gray, with random coiled segments of the polypeptide chain as open (white) coils. The β-strands of β-sheet A are numbered, in white. Note that the reactive centre loop (RCL) inserts between β-strands 3 and 5 as new strand 4 on complex formation with a cognate proteinase. The RCL is shown in blue, with the P (proximal) side in light blue, and the P′ (distal) side in dark blue. RCL residues between P7 and P1′ are shown as sticks to highlight their side chains and numbered in the image; the Proximal and Distal ends of the RCL are highlighted by arrows.

## References

[1] Scott BM, Matochko WL, Gierczak RF, Bhakta V, Derda R, Sheffield WP (2014) Phage Display of the Serpin Alpha-1 Proteinase Inhibitor Randomized at Consecutive Residues in the Reactive Centre Loop and Biopanned with or without Thrombin. PLoS ONE 9(1): e84491 10.1371/journal.pone.0084491 24427287 PMC3888415

