# Supplementary material for: Phage Display of the Serpin Alpha-1 Proteinase Inhibitor Randomized at Consecutive Residues in the Reactive Centre Loop and Biopanned with or without Thrombin
Source: PLoS One. 2014 Jan 10;9(1):e84491. doi: 10.1371/journal.pone.0084491 (PMC3888415; doi:10.1371/journal.pone.0084491)

A.

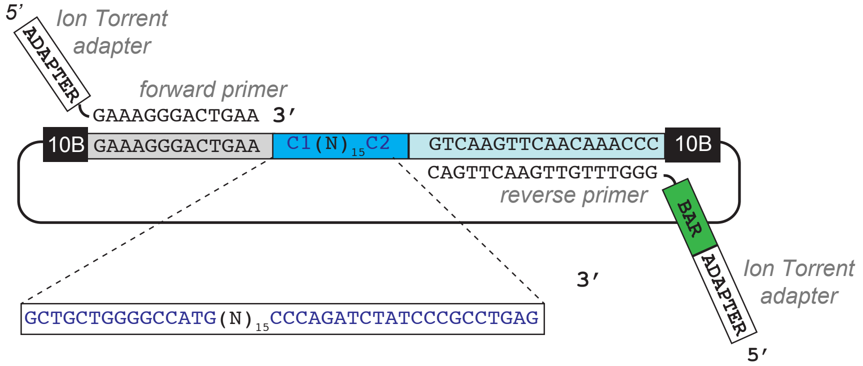

B.

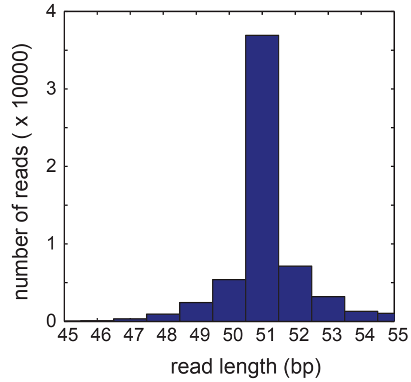

C.

Length	constant region / variable region / constant region	
<51>	GCTGCTGGGGCCATGCTCGACTTTCCTATACCCAGATCTATCCCGCCTGAG	correct read length, correct constant regions
<52>	GCTG**G**CTGGGGCCATGCTCGACTTTCCTATACCCAGATCTATCCCGCCTGA	insertion in the constant region
<52>	GCTGCTGGGGCCATGTCACTCAGATCCCCC**C**CCCAGATCTATCCCGCCTGAG	homopolymer read error
<50>	GCTGCTGGGGCCATGGACGCATTCGCCGCGCC_AGATCTATCCCGCCTGAG	deletion in the constant region (read error)
<50>	GCTGCTGGGGCCATGCTCGACTT_CCTATACCCAGATCTATCCCGCCTGAG	deletion in the variable region (read error)

D.

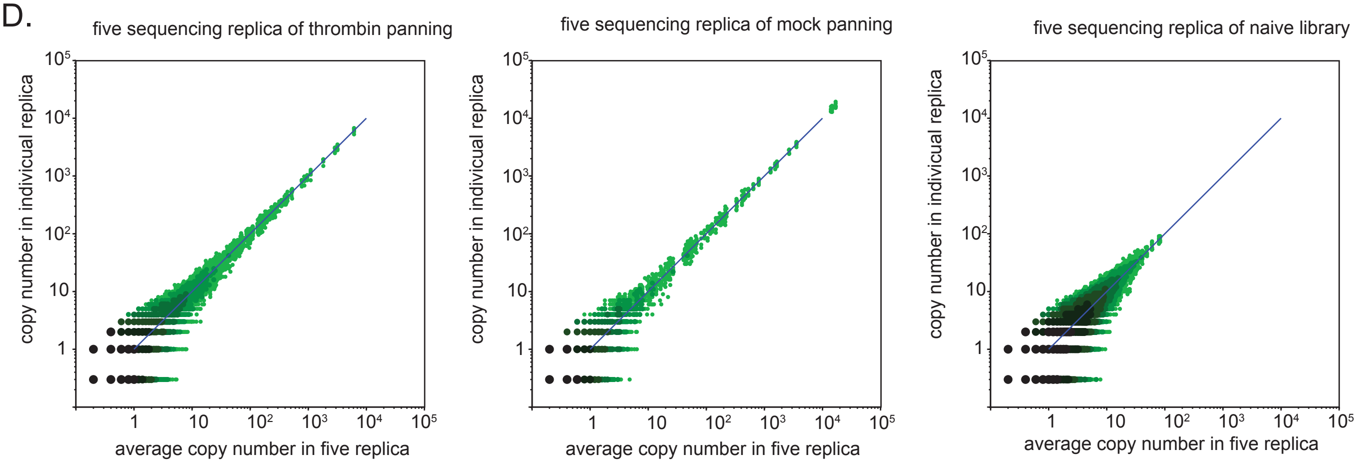

E.

$$X(i) = \sum_{k=1}^{5} \frac{(N(i,k) - M(i))^2}{M(i)} \qquad M(i) = \sum_{k=1}^{5} \frac{N(i,k)}{5}$$

N(i,k) - normalized copy number of seq. i in replica k

M(i) - mean copy number

F.

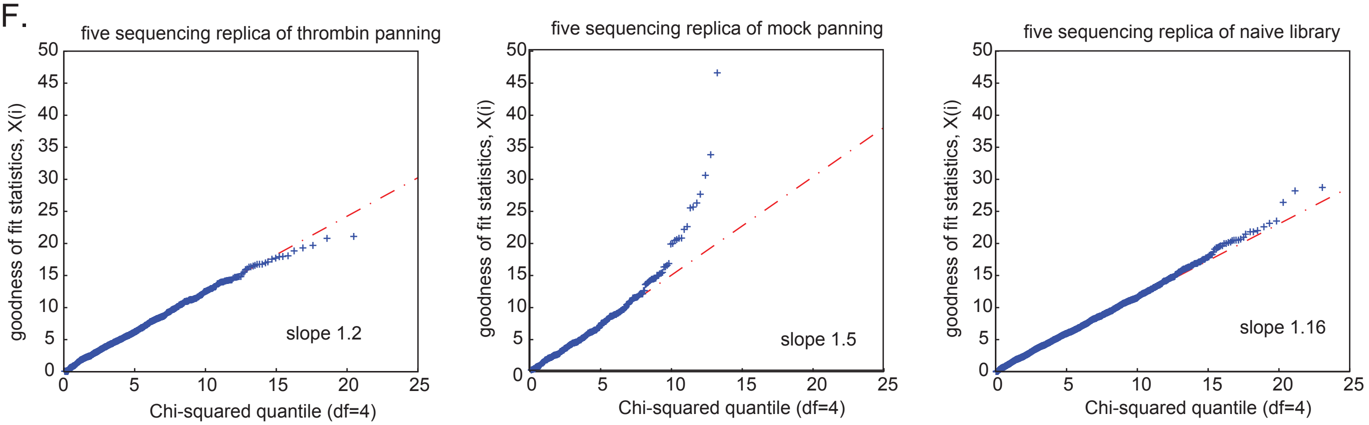

Supplement: Figure S1 — Panel A: Description of the primers used for Ion Torrent sequencing and the structure of the displayed on 10b-displayed insert surrounded by the primer sequences. N is a random nucleotide. Panel B: Representative distribution of read length after Ion Torrent analysis. Panel C: Analysis of the reads of the wrong length revealed insertions/deletions and homopolymer read errors which are common in Ion Torrent reads [60], [61]. Only the reads that contained correct length and correct sequence flanking the (N)15 insert were considered (see Table S2). Panel D: We re-sequenced each library five times and observed that copy numbers were reproducible between the reads. The plots show deviation from an average copy number. Panel E: To show that deviation between sequencing runs follows a noisy Poisson distribution [62], we calculated goodness of fit statistics X(i) for each read i. Panel F: We compared the distribution of X to the Chi-squared distribution with 4 degrees of freedom using a QQ-plot [43]. The thrombin-panned and naïve libraries were found to be normally distributed. The ∼1.2 slope on the QQ-plot indicates that the variance of the reads is 20% higher than the variance of the Poisson distribution (with the increase being due to noise in PCR, emulsion PCR and re-sequencing). In mock panning, a small number of reads deviated from normal distribution; but the majority of the library was found to be normally distributed. As the copy numbers were normally distributed, we used parametric statistics (t-test) to evaluate the enrichment in the screen (see Figure 7). The complete processed deep sequencing data set, showing all (N)15 insert sequences found in this study, is also provided in Table S4 (see below). (PDF) [file pone.0084491.s001.pdf]
