# Supplementary material for: Phage Display of the Serpin Alpha-1 Proteinase Inhibitor Randomized at Consecutive Residues in the Reactive Centre Loop and Biopanned with or without Thrombin
Source: PLoS One. 2014 Jan 10;9(1):e84491. doi: 10.1371/journal.pone.0084491 (PMC3888415; doi:10.1371/journal.pone.0084491)
Supplement: Table S1 — List of Barcodes used in Ion Torrent primers. The DNA sequence of the 18 different barcodes used in Ion Torrent oligodeoxyribonucleotide primers is listed, in standard 5′ to 3′ orientation. (DOC) [file pone.0084491.s002.doc]

| **Number** | **Barcode Sequence (5’ – 3’)** |
| --- | --- |
| 1 | CTAAGGTAAC |
| 2 | TAAGGAGAAC |
| 3 | AAGAGGATTC |
| 4 | TACCAAGATC |
| 5 | CAGAAGGAAC |
| 6 | CTGCAAGTTC |
| 7 | TTCGTGATTC |
| 8 | TTCCGATAAC |
| 9 | TGAGCGGAAC |
| 10 | CTGACCGAAC |
| 11 | TCCTCGAATC |
| 12 | TAGGTGGTTC |
| 13 | TCTAACGGAC |
| 14 | TTGGAGTGTC |
| 15 | TCTAGAGGTC |
| 16 | TCTGGATGAC |
| 17 | TCTATTCGTC |
| 18 | AGGCAATTGC |
