# Supplementary material for: Phage Display of the Serpin Alpha-1 Proteinase Inhibitor Randomized at Consecutive Residues in the Reactive Centre Loop and Biopanned with or without Thrombin
Source: PLoS One. 2014 Jan 10;9(1):e84491. doi: 10.1371/journal.pone.0084491 (PMC3888415; doi:10.1371/journal.pone.0084491)
Supplement: Table S2 — Number of reads identified by Ion Torrent sequencing for different experiments. On the 9 column by 5 row table, “+IIa” indicates selection with thrombin (IIa), “-IIa” selection without IIa, and “N” indicates the naive P7–P3 randomized library; r1, r2, and r3 refer to different deep sequencing replicas. Only three out of five replicas are shown here (but see additional Supporting Information listed below). In the rows, “Total” indicates the total number of reads identified for a specific barcode; “match” refers to the number of reads that match to PCR primers; N55 identifies the number of reads that have the correct insert length; N15 refers to the number of reads that reflect the correct structure of the library; and “Unique” corresponds to the number of unique sequences. (DOC) [file pone.0084491.s003.doc]

| Name | +IIa (r1) | +IIa (r2) | +IIa (r3) | -IIa (r1) | -IIa (r2) | -IIa (r3) | N (r1) | N (r2) | N (r3) |
| --- | --- | --- | --- | --- | --- | --- | --- | --- | --- |
| Total | 134716 | 137390 | 137475 | 102849 | 125517 | 111125 | 130038 | 142872 | 146033 |
| match | 58479 | 61004 | 59761 | 60394 | 65245 | 57797 | 53223 | 58315 | 58724 |
| N55 | 36796 | 38922 | 37427 | 47739 | 49057 | 43782 | 28723 | 32386 | 32810 |
| **N15** | **33778** | **35970** | **34570** | **46321** | **47210** | **42287** | **26615** | **30043** | **30451** |
| Unique | 3644 | 3756 | 3721 | 619 | 725 | 688 | 10054 | 10479 | 10637 |
